# Pulmonary Hypertension and Indicators of Right Ventricular Function

**DOI:** 10.3389/fmed.2016.00023

**Published:** 2016-06-03

**Authors:** Célia von Siebenthal, John-David Aubert, Periklis Mitsakis, Patrick Yerly, John O. Prior, Laurent Pierre Nicod

**Affiliations:** ^1^University of Lausanne, Lausanne, Switzerland; ^2^Pneumology, Centre Hospitalier Universitaire Vaudois, Lausanne, Switzerland; ^3^Nuclear Medicine and Molecular Imaging, Centre Hospitalier Universitaire Vaudois, Lausanne, Switzerland; ^4^Cardiology, Centre Hospitalier Universitaire Vaudois, Lausanne, Switzerland

**Keywords:** pulmonary hypertension, right ventricle, PET/CT, echocardiography, cardiac catheterization

## Abstract

Pulmonary hypertension (PH) is a rare disease, whose underlying mechanisms are not fully understood. It is characterized by pulmonary arterial vasoconstriction and vessels wall thickening, mainly intimal and medial layers. Several molecular pathways have been studied, but their respective roles remain unknown. Cardiac repercussions of PH are hypertrophy, dilation, and progressive right ventricular dysfunction. Multiple echocardiographic parameters are being used, in order to assess anatomy and cardiac function, but there are no guidelines edited about their usefulness. Thus, it is now recommended to associate the best-known parameters, such as atrial and ventricular diameters or tricuspid annular plane systolic excursion. Cardiac catheterization remains necessary to establish the diagnosis of PH and to assess pulmonary hemodynamic state. Concerning energetic metabolism, free fatty acids, normally used to provide energy for myocardial contraction, are replaced by glucose uptake. These abnormalities are illustrated by increased ^18^F-fluorodeoxyglucose (^18^F-FDG) uptake on positron emission tomography/computed tomography, which seems to be correlated with echocardiographic and hemodynamic parameters.

## Introduction

Pulmonary hypertension (PH) is a serious disease, which evolves progressively to cardiac insufficiency and death (1, 2). It is characterized by increased pressures and resistances in pulmonary arteries. The diagnosis is made, by cardiac catheterization, by a mean pulmonary arterial pressure (PAPm) ≥25 mmHg. We talk about pulmonary arterial hypertension (PAH) when the pulmonary wedge pressure (PWP) is ≤15 mmHg and pulmonary vascular resistance (PVR) >3 WU (3, 4). We can classify the etiologies of PH in five different groups, according to DanaPoint classification (2008) and maintained with minor modifications after the fifth World symposium in Nice (2013), with the first group related to etiologies leading to PH of arterial origin and sharing similar histological pattern. All groups of PH are characterized by similar symptoms, especially dyspnea, cough, angina, fainting, or peripheral edemas (4, 5).

Without treatment, survival does not exceed 2–3 years with chronic PAH. Moreover, the diagnosis is usually made when cardiac repercussions and clinical symptoms are already severe [75% of patients in New York Heart Association (NYHA) class III or IV] (1, 5). Thus, it is necessary to develop new tools to make earlier diagnosis and to increase knowledge in medical caregivers about this poorly known disease.

The aim of this article is to review current knowledge about pathophysiology and cardiac repercussions of PAH, as well as the different clinical, biological, and imagery parameters used to assess PAH and right heart function.

## Classification of Pulmonary Hypertension

Etiologies and forms of PH are classified in five groups, according Dana Point classification (2008). Different forms of PAH are joined in Group 1, to know idiopathic PAH, heritable PAH, PAH related to drugs and toxins, connective tissue disease, HIV, schistosomiasis, portal hypertension, and congenital heart disease. As previously mentioned, PWP is <15 mmHg, and PVR is >3 WU. Treatments for PAH are described below. Second group is PH related to left heart disease. Echocardiography and cardiac catheterization show left ventricular dysfunction. PWP is >15 mmHg, and PVR is >3 WU. These forms are mainly treated with usual treatments of left ventricular dysfunction or repair valvular diseases. If these are not sufficient, specific treatments for PH, such as vasodilators, can be added. Third group contains PH forms related to lung disease, such as chronic obstructive pulmonary disease, interstitial diseases, or sleep apneas. They are characterized by a PWP <15 mmHg and pathological pulmonary functional tests. First line treatment is an optimal therapy of the underlying disease and long-term oxygen therapy. Fourth group is chronic thromboembolic PH. As in PAH, PAPm is >25 mmHg, and PWP is <15 mmHg. Multiple thrombi can be found by pulmonary computed tomography or magnetic resonance angiography or by pulmonary ventilation/perfusion scan. Chronic thromboembolic PH can be reversed by endarterectomy, and long-term anticoagulation is recommended. In inoperable patients, or if PH persists after surgery, treatments of PAH can be useful. Last group contains PH forms from multifactorial and unclear mechanisms, such as hematologic diseases, sarcoidosis, or metabolic disease. PWP is a variable (3). After the fifth symposium in Nice (2013), minor modifications have been done. In Group 1, a few more mutations are described. Persistent PH of the newborn has been separated from other causes of PAH and some congenital cardiopathies have been added to Group 2 (4). The aim of this article is to review pathogenesis and evaluation of PAH forms.

## Pathobiology and Therapeutic Treatment Options

### Histology

**Figure 1:**
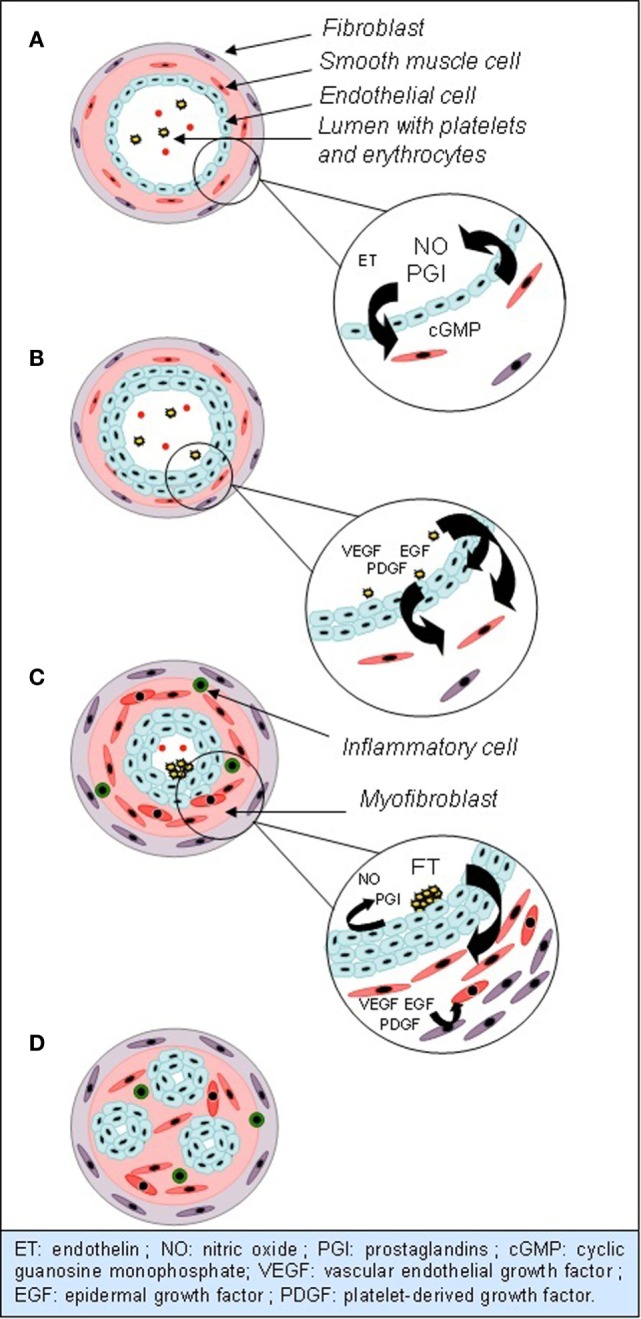
**Evolution of anatomical and functional abnormalities in small arterioles in PAH**. **(A)** Normal vessel: vasodilation is predominant, by NO and PGI. **(B)** Hypertrophy and hyperplasia of endothelial cells, mediated by growth factors. **(C)** Hypertrophy and hyperplasia of smooth muscle cells and fibroblasts. Extravasation of inflammatory cells. Endothelial dysfunction and clots formation. **(D)** Plexiform lesions.

Multiple abnormalities are found in histological analysis of lungs from PAH patients, mostly in small arteries. They all induce thickening of vessels wall. The first step in this process is thickening of intima, by increased proliferation and migration of local stem cells. Medial and adventitial layers are also altered, by medial smooth muscle cells hypertrophy, which invade the wall of small arteries, usually not muscularized, and by accumulation of fibroblasts from the adventitia. Extracellular matrix is produced and degraded in excess by metalloproteinases, which releases mitogenic factors for smooth muscle cells. Eventually, adventitial mesenchymatous cells called myofibroblasts migrate to the media and induce proliferation of smooth muscle cells. Later in the obstructive vascular process, hypertrophy and hyperplasia of endothelial cells, also known as neointima formation, continue and form plexiform lesions. Plexiform lesions are a monoclonal proliferation of endothelial cells forming multiple channels into one obliterated arteriole, surrounded by smooth muscle cells, extracellular matrix, and myofibroblastes (Figure 1). Beyond morphological abnormalities, they present many functional and metabolic alterations (6–8). As in many other proliferative diseases, several growth factors, such as vascular endothelial growth factor (VEGF), platelet-derived growth factor (PDGF), or epidermal growth factor (EGF), are involved in vessels wall hyperplasia, through tyrosine kinase activation. Their overexpression in endothelial cells promotes inflammation, proliferation, migration, and survival of smooth muscle cells and fibroblasts. Greater serotonin sensitivity and increased transporter’s expression were found in smooth muscle cells, and a greater amount of serotonin, through its mitogenic effects, could take part to thickening of vascular wall (6, 7, 9, 10). The trigger for these changes in unknown, but some studies hypothesized that an endothelial injury could promote this proliferation. Indeed, Sakao et al. showed that inhibition of VEGF receptors and mechanical stress can provoke endothelial lesions by apoptosis. After a few days, resistance against apoptosis appears in surviving cells, probably induced by release of mediators from previously injured cells. Resistant cells express survivin, an apoptosis inhibitor frequently met in malignant tumors (11, 12). Parallel to hyperplasia of vessels wall, multiple microthromboses are frequently found in small arteries of PH lungs. Three components promote thromboses: activation of intravascular coagulation, platelets dysfunction, and release of vasoconstrictor and pro-coagulant mediators by endothelial cells. Platelets dysfunction and dysfunctional endothelium promote intravascular coagulation by releasing plasmatic pro-coagulants (von Willebrand factor, thromboxane, and serotonin) and anti-fibrinolytic molecules (plasminogen activator inhibitor). Platelets also store vasoconstrictor and mitotic substances. However, it is unclear if microthromboses are a consequence or a trigger for elevated PVRs and pressures (7–9).

### Endothelial Dysfunction

Many studies identified the nitric oxide (NO) pathway to be linked to the emergence of PAH. NO is produced by NO synthase (eNOS) in endothelial cells. It is the predominant factor inducing vasodilation in normal conditions, *via* cyclic guanosine monophosphate (cGMP). It also inhibits proliferation of smooth muscle cells, platelets aggregation and activation, leukocytes migration, and endothelin (ET) secretion (9, 12). In mice, an inhibiting mutation of eNOS induces increased pulmonary arterial pressure (PAP) (13). In humans, it seems that a reduced amount of NO is also involved in pathogenesis of PAH, by increased degradation and reduced eNOS expression in pulmonary tissues (Figure 1). Giaid and Saleh showed a negative correlation between eNOS expression in immunohistochemical analysis, severity of vascular lesions and PVR (14).

Pathogenesis of PAH can be partly explained by increased ET secretion. ET is a neuropeptide released by vascular endothelium. It can bind, on vascular wall, to type A receptors, leading to vasoconstriction, or type B receptors, leading to vasodilation. In pathological conditions, vasoconstrictor effect is predominant and takes part to PAH development. ET seems to be acting partly by inhibition of Kv1.5 potassium channels synthesis. Increased potassium rate in cells induces depolarization and calcium influx through voltage-dependant calcium channels. Increased intracellular calcium and potassium rates promote vasoconstriction, proliferation, and apoptosis resistance. ET is thus responsible of both hyperplasia and endothelial dysfunction (Figure 1) (9, 10, 15).

Prostaglandins (PGI) pathway could also play a role in PAH development. PGI are derived from arachidonic acid and inhibit cellular proliferation, inflammation, coagulation, platelets aggregation, and vasoconstriction. A reduced rate is found in PAH patients (Figure 1) (7, 9, 12, 16).

This endothelial dysfunction is the target of current PAH treatments, such as ET receptors antagonists, PGI derivates, phosphodiesterases inhibitors, calcium-channel blockers, or cGMP kinase activators. However, it seems that remodeling and occlusion of small arteries contributes primarily to the increase of PAP and right atrial pressure (RAP). Thus, the therapeutic response is highly variable, frequently insufficient, and these treatments cannot reverse the underlying histological abnormalities (3, 8, 9, 15).

### Inflammation and Immunity

Several studies identified an inflammatory component in pathogenesis of PAH. Indeed, different types of inflammatory cells are found on the surface and into the vessels wall. Furthermore, several cytokines and chemokines are excessively produced, by inflammatory and endothelial cells, and also take part to smooth muscle cells proliferation. Soon et al. demonstrated that blood levels of some cytokines, such as interleukin (IL)-2, IL-6, IL-8, or IL-10, could be correlated with survival, which support their implication in pathogenesis of PH. For many years, the presence of autoantibodies has been discovered in PH related to connective tissue disease and systemic sclerosis. Recent studies have also brought them out in idiopathic PAH cases. Indeed, immunoglobulin G (IgG) not only against endothelial cells components but also against fibroblasts and smooth muscle cells is found in about 60% of patients. Although little is known about the role of those antibodies, they could cause endothelial injuries and then trigger the excessive proliferation process (6, 10, 17–19).

### BMPR2 and TGF-β

Mutations of bone morphogenetic protein receptor type II (BMPR2), a kinase receptor from transforming growth factor-beta (TGF-β) family, are not only found in 70% of patients with heritable PH but also in about 25% of patients with idiopathic PAH. BMPR2 is usually expressed in pulmonary endothelial cells and regulates cell growth, differentiation, and apoptosis, through a factor named Smad. Some studies showed that inactivation of BMPR2 promotes endothelial cell survival, proliferation, angiogenesis, and migration. A loss of BMPR2 could be one of the bases of PAH pathogenesis, leading to endothelial injuries, apoptosis resistance, and plexiform lesions. It could also be involved in remodeling and dysfunction of the right ventricle (RV). However, prevalence of PAH in patients with BMPR2 mutation is low, which suggests that those mutations remain predisposing factors and that other vascular injuries are necessary to develop the disease (6, 9, 20). Abnormalities of other members of TGF-β family, such as TGF-β_1_, which induce proliferation and remodeling of vascular wall, seem to be present. Thus, there could be an imbalance between BMPR2 and TGF-β_1_, in favor of an excessive proliferation (16, 20).

### Survivin

Those different pathways have a common consequence: excessive proliferation and apoptosis resistance. As mentioned previously, survivin is involved in some proliferative diseases. McMurtry et al. showed that its expression is increased not only in pulmonary vascular wall, mainly in smooth muscle cells, but also in endothelium and fibroblasts. Survivin inhibits apoptosis and opening of Kv1.5 potassium channels. In rats, survivin inactivating treatments are useful to reduce wall remodeling, PAP, and RV hypertrophy. It seems that survivin expression can occur after endothelial injuries, which is consistent with results obtained about the signaling pathways presented above. This factor could be one of the links between those pathways and vascular wall remodeling (15).

### micro-RNAs

Recent studies in rats showed that another link between these pathways could be related to micro-ribonucleid acids (miRNAs). miRNAs are a family of non-coding RNAs, which regulate expression of coding RNAs through binging and cleavage in the cytoplasm. Different mechanisms, such as hypoxia, hypoxia-inducible factor-1alpha (HIF-1α), inflammation, and growth factors, regulate miRNAs. It seems that the expression of miRNAs could be dysregulated in PAH, as some are increased and others decreased. We can mention the example of miR-204, which regulates cellular proliferation and apoptosis through B-cell lymphoma-2 (Bcl-2) and nuclear factor of activated T-cells (NFAT). This one seems to be decreased in smooth muscle cells. ET and PDGF could be involved in inhibition of miR-204 (21). Other miRNAs are probably involved in PH and right heart dysfunction (22, 23).

### HIF-1α and Metabolism

Several studies demonstrated that endothelial cells and myocardial energetic metabolism is altered. Free fatty acids oxidation, used as energetic resource in normal conditions, is replaced by anaerobic glycolysis. Molecular analysis of endothelial and myocardial cells showed that this metabolic shift is based on normoxic activation of the transcription factor HIF-1α (24–26). This factor is normally activated in hypoxic conditions, in order to produce energy without oxygen, by anaerobic glycolysis. HIF-1α slows down Krebs cycle. Free fatty acids oxidation is thus replaced by aerobic or anaerobic glycolysis. The latter is predominant, explaining increased lactate production, which is observed in PAH. This is called Warburg effect and is found in several pathologies, especially in malignant tumors (24–28). This results in decreased energy production per glucose molecule and, thus increased glucidic needs. Adequate intake is provided by increased expression of glucose transporter 1 (GLUT-1) channels and hexokinase, which are found in greater amount, particularly in pulmonary vessels with plexiform lesions (24, 25). In rats and in humans, Bonnet et al. showed that all those elements reduce cellular metabolic dependence to oxygen and give an advantage in hypoxic conditions for cellular proliferation and survival. This process depends on the inhibition of Kv1.5 potassium channels by HIF-1α. The resulting cellular depolarization induces calcium inflow. Elevated intracellular potassium and calcium rates promote proliferation and apoptosis resistance (29). In pulmonary vessels, parallel to this metabolic shift, the reduced NO production observed in PAH leads to a decreased number of mitochondria per cell and inhibits mitochondrial genes expression. Mitochondrial function abnormalities are also found, specifically in PAH lungs. This results in a reduced production of reactive oxygen species and a decreased amount of superoxide dismutase (SOD). Hydrogen peroxide production (H_2_O_2_), normally responsible of HIF-1α inhibition, is inhibited. These signaling alterations by reactive oxygen species could be the basis of normoxic activation of HIF-1α (24, 26, 28, 29). In the myocardium, inhibition of aerobic metabolism probably takes part to development hypertrophy, in which a greater expression of GLUT-1 channels, decreased oxygen consumption and increased lactate production by anaerobic glycolysis are found. This could lead to reduced energetic reserves and further reduce right heart function (24, 26, 30). Experimental studies investigated the efficacy of treatments targeted on this metabolic shift, such as imatinib or dichloroactetate. They demonstrated beneficial effects on pulmonary remodeling, inflammation, and myocardial hypertrophy (24, 25, 27). Feasibility and usefulness of these treatments in humans remain to be determined.

### Conclusion

In conclusion, several mechanisms have been proposed to explain the abnormal pulmonary vasoconstriction and hyperplasia, but links between these different pathways remain unclear. Current treatments are not always effective and cannot reverse the histological abnormalities. Experimental studies are looking for new therapeutic targets, in order to inhibit proliferation, reactivate apoptosis, and recover a normal organization in pulmonary vascular wall.

## Right Ventricular Physiopathology

### Hypertrophy and Dilation

In normal conditions, the RV is a complex structure with three different compartments: the filling chamber with the tricuspid valve, the apical chamber with the right ventricular free wall, and the outflow tract, near from the pulmonary valve. These three chambers play a different role in blood ejection toward the pulmonary artery (31). In PAH, the RV loses its contraction delay, which is necessary for blood diffusion through the ventricle toward the pulmonary artery. All the ventricular structure is altered, and the three-chambered structure evolves to a more cylindrical shape, which allows blood ejection despite simultaneous contraction of all compartments. Those contraction disturbances of ventricular wall can be explained by abnormalities of myocardial contractility, secondary to an alteration of muscular fibers orientation (31, 32).

In normal conditions, Kind et al. observed that the interventricular septum is made of oblique- and helical-shaped fibers, leading to a twisting contraction. In contrary, right ventricular free wall is mostly made of transverse fibers, leading to a circumferential contraction. In PAH, septal fibers orientation becomes transverse, which promotes a circumferential contraction, observed in the right ventricular remodeling (33).

When the afterload increases because of PAP elevation, RV hypertrophy maintains a normal RV ejection fraction (RVEF) and diminishes wall stress (34, 35). However, even if this mechanism first preserves cardiac function, it leads to diastolic dysfunction, ischemia, and dilation of the RV and is, furthermore, not sufficient to preserve cardiac function. Indeed, after pressure overload occurring in the beginning of PAH, a volume overload appears, probably secondary to tricuspid regurgitation, although all mechanisms are not well known yet. This dilation is mainly responsible of dysfunction of the RV (36–38). According to Archer et al., we can still differentiate two different phenotypes, depending on cardiac adaptation to pressure elevation: the adaptive phenotype, where the RV is less dilated and fibrotic, the RVEF is preserved and remodeling is concentric, and the maladaptive phenotype, where the RV is dilated, fibrotic, hypokinetic, and remodeling is eccentric (24). Little is known about the mechanisms leading to one or the other phenotype, but adaptive phenotype is rather observed in children with Eisenmenger syndrome, while maladaptive phenotype is seen in other types of PAH. There is also probably a continuum between these two patterns, as the elevated PAP becomes greater and perpetual (34). Indeed, studies showed a good correlation between cardiac remodeling and function, and pulmonary vascular abnormalities and hypertrophy. This has led to the conception of RV and pulmonary circulation as a unit, where one component cannot be considered alone, in order to get a better comprehension of their pathophysiology and relations (39–41).

### Histology

It is well known that, following exposition to elevated PAP, the RV becomes hypertrophic. Histological examination of PAH hearts has demonstrated that the myocytes diameter is increased, in comparison with normal hearts. It seems that increased right ventricular mass is also secondary to cytoplasmic proteins and sarcomeres production (41, 42). Moreover, it has been demonstrated that the passive tension of sarcomeres is increased, by reduced phosphorylation of titin, which usually regulates tension of sarcomeres. This induces an impaired relaxation of myocytes. In parallel, deposition of collagen in the extracellular matrix induces fibrosis of the myocardium. Both impaired relaxation of myocytes and collagen deposition increase rigidity of the RV and induce a diastolic insufficiency, which could participate to the right ventricular dysfunction (37). Even if the myocardium has limited regenerative and proliferative properties, it seems that hyperplasia, by proliferation of progenitor cells, add to hypertrophy. After hypertrophy has occurred, these changes of structure and function of myocytes, followed by increased apoptosis, seem finally to lead to contractile dysfunction and dilation of the RV (38, 41, 42). Pathways determining those changes are not well known yet. However, it seems that they are probably triggered and maintained by inflammation, by neutrophils and ILs, oxidative stress, systemic and local neurohormonal factors, and ischemia (38, 42, 43). Despite many structural abnormalities happen, it seems that these modifications are, at least partially reversible, as it is well known that cardiac function improves after lungs transplantation or endarterectomy (42).

In the myocardium, alterations of energetic metabolism, similar to pulmonary vessels, participate to right ventricular hypertrophy and dysfunction. They lead to decreased oxygen consumption, increased lactate production, and reduced energetic reserves. Increased GLUT-1 channels and HIF-1α activation are found in PAH myocardium and seem to be associated with greater right ventricular dysfunction (24, 26, 30).

### Septum and Left Ventricle

Interventricular septum acts like a passive membrane during diastole and systole, whose position depends on blood volume and pressure in the left ventricle (LV) and the RV. In PAH, when the right ventricular pressure becomes greater than the left ventricular pressure, septum is moved leftward, according to pressure gradient. We talk about abnormal eccentricity when the septum is moved leftward during systole (44, 45). The septum plays an important role in both ventricular contraction and even more in the RV in PAH. Indeed, according to López-Candales, it seems to be the “ventricular motor” in the right ventricular contraction against elevated afterload, which means that it provides most of the strength needed for blood ejection. Septal deviation is thus involved in progression of right ventricular dysfunction (45).

Right heart dysfunction, by several mechanisms, can also lead to a left cardiac dysfunction, related to interdependence of both ventricles. Septal abnormal eccentricity, as previously described, induces an abnormal left ventricular morphology. Furthermore, as the pericardium is not much extensible, the hypertrophied RV can compress the LV and diminish its volume. This results in left cardiac dysfunction, characterized by a reduced left ventricular filling, responsible for a diminished ejection volume (34, 45).

Finally, in PAH, we can find a dyssynchrony between both ventricles: right ventricular free wall is still contracting when the diastole has begun in the LV, which accentuates septal deviation and shortens diastole in the RV. Thus, filling is also reduced in the RV (32, 34, 46).

### Blood Flow

Cardiac perfusion has been mostly studied in left cardiac hypertrophy. In the left coronary artery, in normal conditions, blood flow depends on arterial pressure, or aortic pressure, myocardial capillary pressure, determined by several neurohumoral and metabolic factors, and left ventricular pressure (47). In normal conditions, blood flow in the right coronary artery does not depend on ventricular contraction. It is a monophasic flow, which means that systolic flow is equal to diastolic flow. In contrary, in PAH, van Wolferen et al. demonstrated that the right coronary blood flow was diminished during systole, and increased during diastole. First, blood flow becomes biphasic, as seen in the left coronary artery in normal conditions, and thus depends on contraction and right ventricular systolic pressure (48). Second, the systolic coronary blood flow per mass unit decreases parallel to the progression of right ventricular hypertrophy, because of microvascular rarefaction compared to ventricular mass and by vessels compression during systole. This can explain the presence of sub-endocardial ischemia in severe right ventricular hypertrophy, as it is found in left ventricular hypertrophy (24, 38).

## Clinical Parameters

### Cardiopulmonary Exercise Test

Cardiopulmonary exercise test (CPET) has been widely used to assess exercise capacity in PAH patients. The patient undergoes ergometry with defined initial load, which is progressively increased until maximal symptom-limited intensity is reached. Several variables are measured: oxygen pulse which is oxygen consumption per heartbeat, maximal oxygen consumption (V_O2_), ventilatory equivalent for carbon dioxide (V_E_/V_CO2_), which reflects respiratory adaptation to exercise and carbon dioxide (CO_2)_ production, heart rate, arterial oxygen saturation (SaO_2_), and systemic arterial tension. It has been showed that V_O2_ is decreased in PH and, as it reflects maximal cardiac output (CO), is a marker of impaired cardiac function. Indeed, several studies showed that decreased V_O2_ and increased V_E_/V_CO2_ are correlated with cardiac function. CPET seems to be a good test to evaluate functional capacity, as it is correlated with NYHA class, disease severity, clinical worsening, and survival in PAH. It is thus suitable for risk stratification. Studies also showed that it is a good test to evaluate treatment response. However, CPET is not suitable for most severe forms of PH and its realization needs technical expertise (49–52).

### Six-Minute Walk Test

The six-minute walk test is a submaximal exercise test, frequently used in assessment of cardiovascular and pulmonary diseases, especially to measure the response to a medical intervention. The patient must walk during 6 min, choosing his walking velocity. He can use any walking help he usually needs and must not interrupt his medication. Dyspnea and tiredness are evaluated, before and after the test, with Borg scale. Even though the patient must provide a physical exertion, the test is well tolerated, even for severely disabled patients, because they can choose intensity and velocity of their walk and can stop at any time. Several contraindications must be taken in consideration, such as unstable angina or infarction in the past month. Incidence of complications, such as arrhythmias or cardiac arrest, is, for now, unknown, and it is thus necessary to perform this test in a place where emergency intervention is possible, where rapid administration of drugs and oxygen is feasible, and under surveillance of a physician certified in cardiopulmonary resuscitation (53).

Several studies demonstrated that it is a good, reproducible test to assess patients’ functional capacities. It represents the physical activity of daily living and thus estimates patients’ capacities in a more reliable way than with a self-reporting questionnaire. Although physical exertion is not maximal, it has been demonstrated that the results are correlated with maximal exertion tests (49). In PAH, results are strongly correlated with survival, quality of life, NYHA class, and hemodynamic parameters. For evaluation of treatment benefits, a modification of walk distance superior to 33 m is considered to be significant, even though the correlation between walk distance and survival remains controversial. However, walk distance reflects the global cardiovascular, pulmonary, and neuromuscular state and is thus not specific to the investigated disease. Despite a weaker sensitivity to assess treatment response in comparison with other exercise tests, it seems that, thanks to its good reproducibility and easy execution, it is most suitable to assess evolution of functional capacities and cardiovascular state after treatment (2, 5, 49–52, 54).

### NYHA Class

New York Heart Association class is a self-assessment of dyspnea by the patient. This classification seems to be a good indicator of survival in PAH: survival is about 6 years in class I and II, 2.5 years in class III, and 6 month in class IV. Some studies showed a good correlation between NYHA class and hemodynamic parameters. Furthermore, treatment-induced changes can be easily measured and are also predictive of survival. Thus, NYHA class could be used to adapt treatment (5, 52, 55, 56). However, this classification has some limitations, such as important interobservers variability and the fact that it is a subjective and self-reported evaluation. Moreover, this classification lacks of sensitivity, as it is difficult to use it to detect precociously patients with PAH. Detection of improvement from a class II to a class I is difficult as well (52).

### Equations

Eventually, in clinical practice, in order to improve patients’ classification according to their prognosis, equations combining different clinical, biological, and hemodynamic parameters have been developed. As their prognostic value relies on diagnostic and therapeutic advances, these equations must be regularly updated. The most recent equation results from the REVEAL study performed by Benza et al. It seems to allow a better risk stratification, in comparison with variables considered individually (1, 56).

## Biological Parameters

### N-Terminal of the Prohormone Brain Natriuretic Peptide

N-terminal of the prohormone brain natriuretic peptide (NT-proBNP) is a peptide released by myocardium when ventricular wall stress is increased by volume overload. It may be increased by any left or right cardiac pathology leading to reduced cardiac function, and its rate reflects changes of ventricular wall stress. In PAH, it has been demonstrated that a higher rate is associated with reduced survival (52, 54, 56–58). Indeed, Mauritz et al. showed that values above 1.256 pg/ml are associated with a worse prognosis. NT-proBNP rates are correlated with hemodynamic parameters, and negatively correlated with CO and RVEF (58–61). NT-proBNP was also an indicator of a long-term treatment response (61). Park et al. showed that decreased NT-proBNP rates after 3 months of treatment with epoprostenol were associated with a higher survival, particularly if they decreased more than 50% from baseline. Thus, it is a sensitive marker of right ventricular dysfunction’s severity and could allow an early assessment of treatment response (62). NT-proBNP is exclusively cleared by kidneys, and its rates can thus be overestimated in chronic renal impairment, frequently found in PAH. In this case, it seems that the correlation between NT-proBNP rates and hemodynamic parameters could be weaker (57, 58).

### Diffusion Capacity for Carbon Monoxide

Diffusion capacity for carbon monoxide (DLCO) is used in a large number of pulmonary diseases, because it allows non-invasive detection of pathological processes inducing perturbations in gas exchanges in pulmonary vessels. It is frequently used in systemic sclerosis to reveal the presence of PH, as its diffusion is altered by arterial wall remodeling. In this pathology, DLCO is also strongly correlated with prognosis (63). In other types of PAH, although remodeling processes are similar, usefulness of DLCO as a prognostic factor remains controversial. Chandra et al. showed that a value inferior to 40% of predicted value is associated with a worse prognosis. In another study, a reduced DLCO was associated with increased mortality, while values above 80% of predicted were associated with increased survival. In contrary, Trip et al. could not demonstrate any association between DLCO and prognosis or hemodynamic parameters. Reduced DLCO in patients with PAH is correlated with smoking, which could mean that reduced values reflect rather an underlying pulmonary pathology than a severe vascular wall injury. Furthermore, no changes in DLCO were found after treatment (1, 56, 64, 65). In clinical practice, DLCO is a part of pulmonary function tests, which is mostly used in clinical practice in initial assessment of PH to detect connective tissue disease or interstitial lung diseases. However, the other variables of pulmonary function tests have not been included in studies and no correlation has been made with prognosis in PAH.

## Markers of Cardiac Function

### Cardiac Catheterization

Today, right heart catheterization remains the gold standard for the diagnosis of PH. We can measure different hemodynamic parameters, such as RAP, PVR, systolic pulmonary pressure (PAPs), PAPm, diastolic PAP (PAPd), PWP, cardiac index (CI), defined as the ratio between CO and body surface area, or SaO_2_, and venous oxygen saturation (SvO_2_). As previously mentioned, it is used in clinical practice to differentiate pre- and post-capillary PH: PWP < 15 mmHg and diastolic pressure gradient (PAPd-PWP) >7 mmHg suggest pre-capillary PH (3). A lot of studies showed a good correlation between prognosis, survival and RAP, PAPm, SvO_2_, CO, and CI. Correlation between CO and PVR is consistent with the concept of RV-pulmonary circulation unit (1, 2, 44, 52, 56, 66–70). PVR are also strongly correlated with survival, although association between their decrease after treatment and mortality is controversial. However, Sitbon et al. showed that a decrease of 30% or more in comparison with baseline values is associated with a better prognosis (55, 71). Evolution of CO after 16 weeks of treatment is also associated with prognosis, as an increase >0.22 l/min is predictive of a better survival (69). Nickel et al. demonstrated that CO values superior to 2.5 l/min/m^2^, and SvO_2_ superior to 65%, at the time of diagnosis, are associated with increased survival. Nevertheless, it is mainly their evolution that determines the patient’s prognosis: worsening despite satisfying baseline values is associated with a higher mortality rate. In contrary, improvement despite reduced baseline values predicts a lower mortality rate. These results evidence the importance of follow-up values (72). During stress, in normal conditions, CO and CI increase. Adaptation of CI during stress could allow a better stratification of patients, as an increase is predictive of a better survival. A positive correlation also exists with six-minute walk distance, and a negative correlation with NYHA class. This illustrates that, if the heart is capable to increase its output during stress, cardiac function is still preserved (73).

However, cardiac catheterization is not suitable for patients’ long-term follow-up, as it is quite invasive. Furthermore, values are most of the time, obtained at rest and cannot allow evaluation of cardiovascular adaptation to stress.

### Echocardiography

Echocardiography is used as one of the first tool to estimate the presence of PH from any group, mainly by measuring PAPs. Tricuspid gradient is estimated from peak tricuspid regurgitation flow velocity (*V*). Pressure differential (Δ*P*) between the RV and the right atrium (RA) is obtained by Bernoulli equation: Δ*P* = 4*V*^2^. Without pulmonary stenosis, PAPs can then be estimated by adding RAP, obtained from the diameter of the vena cava and its respiratory variation: a diameter of vena cava >21 mm with a collapse <50% with deep inspiration suggests a pathological RAP value (>15 mmHg). According to the guidelines, this value cannot, itself, make the diagnosis of PAH. Indeed, in about 50% of patients, PAPs values estimated with ultrasound do not correspond with values obtained at cardiac catheterization. Furthermore, in certain situations, Bernoulli equation cannot be applied, because pressure gradient is underestimated (3, 74). Diagnosis of PAH should be considered, with a degree of probability, in the presence of several evocating signs at echocardiography. Even if PAP values measured by cardiac catheterization are correlated with survival, there is no evidence demonstrating such association with values obtained at ultrasound. Global evaluation of right and left cardiac chambers, as well as valvular state, helps to establish the probability of a pre- or post-capillary origin for PH.

The presence of right heart hypertrophy and dilation can be assessed with two-dimensional echocardiography (36). As previously mentioned, hypertrophy, even having deleterious effects, is an adaptive mechanism allowing the RV to function despite elevated PAP. It is mostly when right ventricular dilation occurs that right ventricular dysfunction appears. Indeed, it seems that dilation of RV, assessed by echocardiography, is better correlated with survival than hypertrophy. Ghio et al. demonstrated that increased ventricular diameter is associated with higher PVR, a higher RAP, a lower CI, and a worse survival (36).

RV ejection fraction, which reflects right ventricular function, can be measured by echocardiography but remains a visual estimation and is thus unreliable. It is better to use other indicators of ventricular function, which also allows an earlier recognition of right ventricular dysfunction. We can use the tricuspid annular plane systolic excursion (TAPSE). Several studies demonstrated that a reduced TAPSE is correlated with a greater right ventricular dysfunction and a worse survival (35, 70, 75, 76). Forfia et al. determined a cut-off of 1.8 cm below which is predicted a worse prognosis, and greater cardiac remodeling and dysfunction (76). TAPSE only illustrates longitudinal shortening of the RV. It seems that, during progression of right ventricular dysfunction, TAPSE reaches a limit and does not diminish further, even if right ventricular function and ejection fraction (EF) are still worsening. In contrary, transverse systolic shortening, measured by cardiac magnetic resonance imagery (cMRI) or echocardiography, illustrates right free wall and septal motion and is correlated with EF all along the progression of dysfunction. Even though free wall deviation also reaches a limit, septal displacement progresses with dysfunction. A reduced transverse shortening is thus mainly due septal deviation (33, 77). We can combine longitudinal and transverse shortening in one parameter: the right ventricular fractional area change (RVFAC), assessed by cMRI or two-dimensional echocardiography. We calculate RVFAC values through right ventricular end-diastolic and end-systolic areas like this: RVFAC = [(RV end-diastolic area − RV end-systolic area)/RV end-diastolic area]. RVFAC is correlated with RVEF and its evolution through the time and could thus be used as a marker of right ventricular function and its evolution. However, its assessment by two-dimensional echocardiography remains difficult and less sensitive than by cMRI (77, 78).

**Figure 2:**
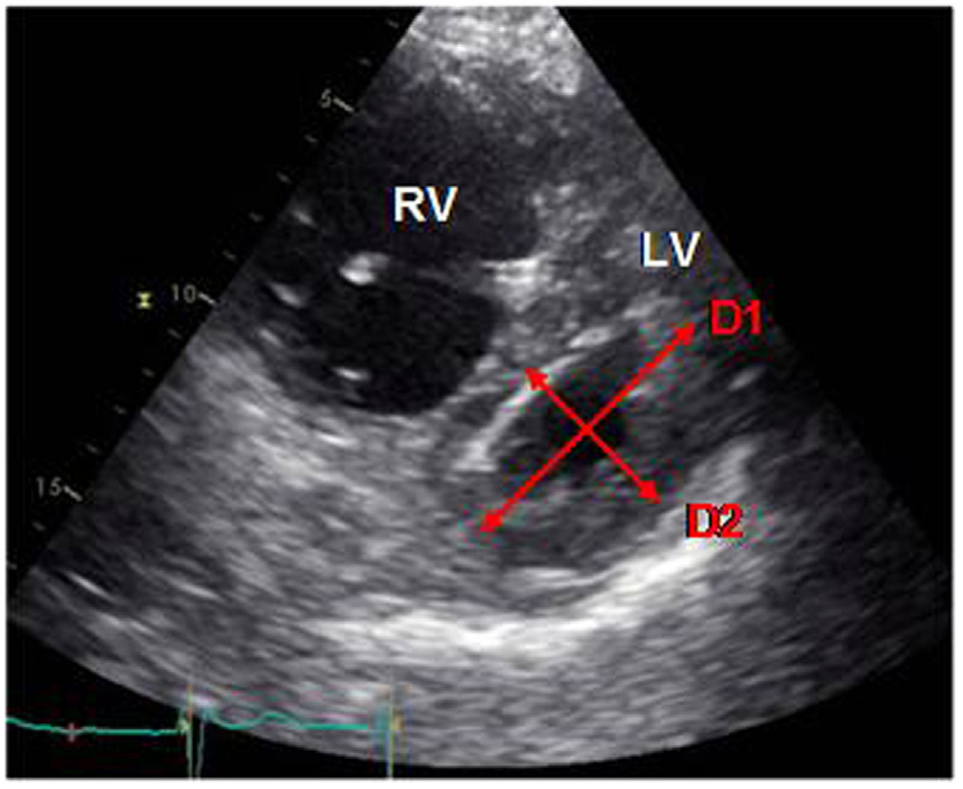
**Measure of the EI (D1/D2) on a short axis view**. D1, anteroposterior diameter; D2, septo-lateral diameter (own unpublished data).

Septal position can also be assessed by the eccentricity index (EI), described first by Ryan et al., which seems to be a marker of both volume and pressure overload in the RV. It is calculated as the ratio between anteroposterior and septo-lateral diameters of the LV, on a short axis view in two-dimensional echocardiography. (Figure 2) (79). Several studies demonstrated that an increased EI, by septal displacement, is correlated with elevated RAP and PAP and is associated with a bad prognosis and a worse survival. The EI seems to be a very good indicator of right ventricular dysfunction and could thus be used as a follow-up tool (44, 45, 80). Moreover, Ghio et al. showed that association of TAPSE and EI allows detection of patients with global, diastolic and systolic dysfunction of the RV (80).

Several studies agree about pericardial effusion to be an excellent predictive factor of mortality (35, 44, 66, 70). As it is due to lymphatic and venous drainage obstruction in the RA, secondary to elevated pressure in this one, it reflects diastolic dysfunction (35, 70).

Tei myocardial index is defined as the sum of isovolumetric contraction and relaxation, divided by ejection time. It is a good indicator of global right ventricular function and patient’s prognosis which is, furthermore, little influenced by ventricle geometry, tricuspid regurgitation, and right ventricular filling state. It is reproducible and easily calculated, by pulsed or tissular Doppler, from pulmonary and tricuspid valves. Increased Tei index is associated with a major right ventricular dysfunction (35, 70, 74).

### Cardiac Magnetic Resonance Imaging

Cardiac magnetic resonance imaging is frequently used in PH to assess morphology and function of the RV. It is not the first tool used to diagnose PH, even if PH should be considered in presence of vascular late gadolinium enhancement and reduced pulmonary arterial distensibility (3). Quite the same morphological and functional parameters as echocardiography are studied with cMRI, i.e., right ventricular diameter and morphology, RVEF, RVFAC, and CO. cMRI can assess more precisely and more easily the complex three-dimensional shape of the RV as well as the RVEF. No surrogates to RVEF are needed, and thus it may be preferred to echocardiography. Moreover, it has been demonstrated that right ventricular diameter and RVEF are correlated with survival (30, 35, 39). It has also a better reproducibility than echocardiography and can be used to assess response to therapies (35, 39, 52). It is also possible to measure blood flow in coronary arteries to detect ischemic abnormalities, which participate to right ventricular dysfunction (52). Right ventricular mass can be easily measured and seems to be correlated with right ventricular function and survival (35, 39). Magnetic resonance angiography can be done simultaneously and can provide information about pulmonary vasculature. Indeed, vascular remodeling can be seen by loss of peripheral vascular enhancement and ramifications of vascular bed. Therefore, MRI allows evaluation of all RV-pulmonary circulation unit (39). However, despite major advantages, fewer studies have been done about usefulness of cMRI in PH evaluation in comparison with echocardiography, and some correlations with other clinical parameters are lacking. cMRI is also expensive and less available than echocardiography and can be limited by claustrophobia and ferromagnetic devices (35).

### Conclusion

In conclusion, in PAH, a lot of markers of right ventricular function have been developed (Table 1). However, their predictive values remain, for most, to be determined. It is recommended to use the best studied markers, such as anatomical parameters, pericardial effusion, or TAPSE (70, 74).

**Table 1:** **Advantages and downsides of parameters for evaluation of PH**.

Parameter	Advantages	Downsides
**CPET** – V_O2_ – V_E_/V_CO2_ CO_2_ – Heart rate – SaO_2_ – Systemic arterial tension	Decreased V_O2_ reflects limitation of maximal cardiac output, marker of impaired cardiac function. Good test to evaluate functional capacity, prognosis, and treatment response	Not suitable for most severe forms of PH needs technical expertise
**6MWT** – Walking distance – Dyspnea – SaO_2_	Well tolerated, even for severely disabled patients. Reproducible test, correlated with maximal exertion tests, survival, quality of life, NYHA class, and hemodynamic parameters in patients with moderate or severe disease	Walk distance reflects the global cardiovascular, pulmonary, and neuromuscular, not specific to PAH alone
**NYHA**	Good correlation with hemodynamic parameters. Evaluation of treatment response, predictive of survival	Important interobservers variability, subjective, and self-reported evaluation. Lacks of sensitivity
**NT-proBNP**	Higher level associated with reduced survival. Correlated with hemodynamic parameters, and negatively correlated with cardiac output (CO) and RVEF. Indicator of long-term treatment response	Increased by any left or right cardiac pathology, overestimated in chronic renal impairment
**DLCO**	Non-invasive detection of perturbations in gas exchanges in pulmonary vessels. Strongly correlated with prognosis in systemic sclerosis	Prognostic value controversial in other forms of PAH. Not modified by treatment
**Cardiac catheterization** – RAP – PVR – PAPs – PAPm – PAPd – PWP – CI – SaO_2_ – SvO_2_	Gold standard for the diagnosis of PH. Used in clinical practice to differentiate pre- and post-capillary PH. SvO_2_ and right filling pressure have a good correlation with prognosis and survival	Invasive, not suitable for patients’ long-term follow-up
**Echocardiographic parameters** – PAPs – Global evaluation of cardiac chambers and vavlulae – RV diameter – RVEF – RVFAC – TAPSE – EI – Tei myocardial index	Easily available, unexpensive. First tool for screening. Many parameters to evaluate global anatomy and function of the RV and LV, as well as valvular state. Correlation with right ventricular function and survival	High intra- and interobservers variability, 3D evaluation of the whole RV difficult. Many parameters remain semi-quantitative. RVEF unreliable, surrogate parameters needed
**cMRI** – Right ventricular diameter and morphology – RVEF – RVFAC – Cardiac output – Blood flow – Magnetic resonance angiography	More precise and accurate evaluation of the complex three-dimensional shape of the RV. No surrogate to RVEF needed. Evaluation of all RV-pulmonary circulation unit: vascular remodeling can be seen by angiography by loss of peripheral vascular enhancement and ramifications of vascular bed. Good reproducibility can be used to assess response to therapies	Fewer studies have been done, some correlations with other clinical parameters are lacking. Expensive and less available than echocardiography, limited by claustrophobia and ferromagnetic devices
**^18^F-FDG-PET/CT (SUV)**	Biomarker of the metabolic shift in the RV and early marker of cardiac dysfunction. Correlated with hemodynamic values, NT-proBNP, several echocardiographic parameters, and long-term prognosis	Not available in every center. Irradiation. Not used in clinical practice yet

## Positron Emission Tomography/Computed Tomography

### ^18^F-FDG Uptake and Energetic Metabolism

**Figure 3:**
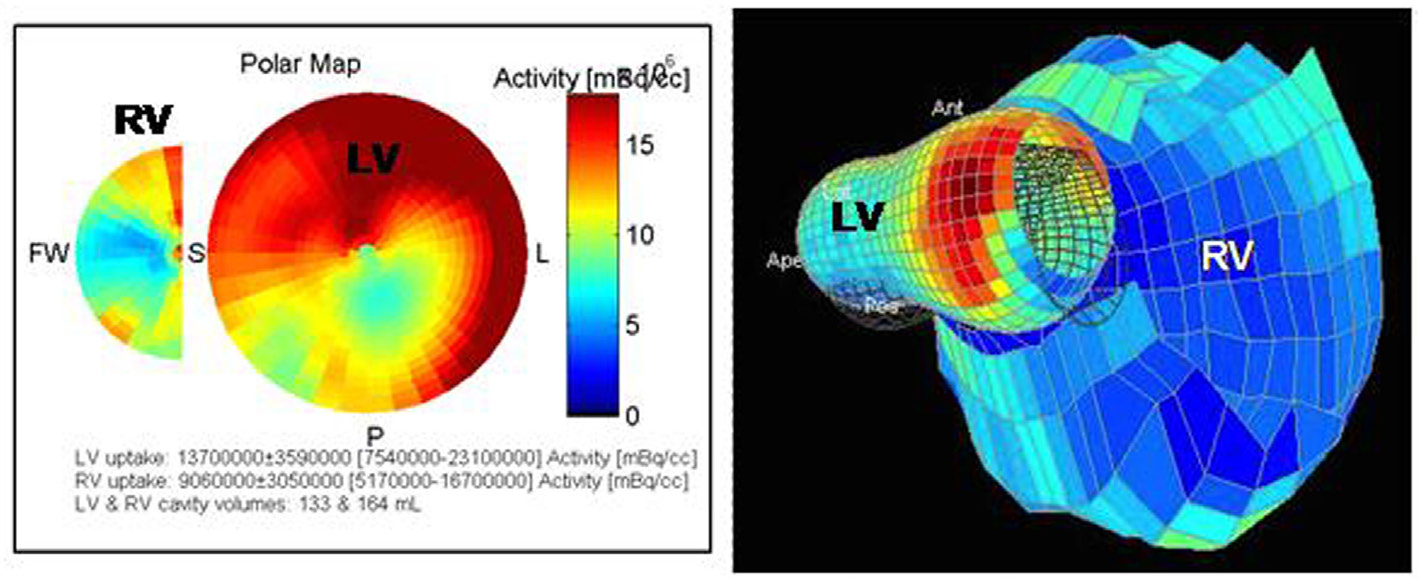
**Pictures obtained from a 67-year-old man with PH of mixed origin (COPD, left heart dysfunction)**. 18F-FDG-PET/CT shows a dilation of the RV with an increased and heterogeneous uptake. A hypometabolism of the infero-lateral medial wall is found in the LV (own unpublished data).

It is possible to evidence these metabolic abnormalities, in rats and in humans, with ^18^F-fluorodeoxyglucose (^18^F-FDG) positron emission tomography/computed tomography (PET/CT), both in myocardium and in pulmonary vessels (Figure 3) (25–27, 81). We usually describe ^18^F-FDG uptake by the standard uptake value (SUV), an index measured as: SUV = tracer concentration in a given place/(injected activity × body mass). In experimental studies, it has been shown that ^18^F-FDG uptake in PAH is secondary to increased GLUT-1 channels expression and anaerobic glycolysis activation (25, 81–83). In the myocardium, increased ^18^F-FDG uptake is also due to a greater cardiac work or myocardial stress, which is the strength that the heart must provide to eject a blood volume against elevated pulmonary pressure. It can also be due to ischemia present in hypertrophy, which can switch the metabolism toward anaerobic glycolysis (81, 84). In contrary, it has been demonstrated that increased uptake cannot be explained by hypertrophy itself or by increased right ventricular free wall thickness (81).

### Severity and Prognosis

A higher mortality rate has been observed when free fatty acids uptake was reduced in favor of glycolysis. Indeed, Tatebe et al. demonstrated that the SUV of the right ventricular free wall is correlated with long-term prognosis in patients with PAH, with a threshold of 8.3, below which survival is decreased. SUV could thus be useful as a biomarker of this metabolic shift and thus, as an early marker of cardiac dysfunction (25, 26, 30, 83). It could allow a better stratification of patients according to the severity of the disease and be used to define best their prognosis. Several studies evidenced that ^18^F-FDG uptake ratio between the RV and the LV is correlated with PAP (30). Kluge et al. demonstrated that RVEF and Tei index are correlated with uptake ratio, by reduced uptake in the LV. This could be explained by decreased energetic needs in the LV because of diminished parietal stress and pre-load (84). In contrary, other studies also showed the same correlation, but explained by increased uptake in the RV (82, 85). ^18^F-FDG uptake is thus correlated not only with hemodynamic values but also with NT-proBNP (85). Lundgrin et al. showed that the right myocardial SUV is correlated with several echocardiographic parameters, such as the right ventricular end-systolic diameter, the EI, and the RFVAC (26). A recent study performed by Li et al. confirmed the relationship between RV/LV ^18^F-FDG uptake ratio and cardiac dysfunction, as an increased uptake was an independent predictive factor of a long-term poor prognosis and mortality. Thus, PET/CT seems to have a prognostic value in assessment of right ventricular function (86).

Oikawa et al. showed that, in patients responding to treatment with epoprostenol, the decrease of right ventricular SUV was correlated with changes of PVR, PAPm, and parietal stress of the RV (81). ^18^F-FDG-PET/CT could also be a useful tool to assess treatment response.

### ^82^Rb-PET/CT

Studies about left ventricular hypertrophy showed that the PET/CT with rubidium-82 (^82^Rb), associated with a stress test and a cold pressor test, is a sensitive and non-invasive exam that can be used to evidence the presence of endothelial dysfunction and perfusion abnormalities in coronary arteries (87–90). However, it has never been used in the context of PH. Endothelial dysfunction has been demonstrated to be a part of the pathogenesis of PAH in pulmonary arteries, but only a few studies evidenced its presence in the systemic and coronary circulations (91–93). In the future, ^82^Rb-PET/CT could be used to study perfusion abnormalities of the RV and to better understand right ventricular dysfunction.

## Conclusion

Despite major discoveries have been made these past years concerning the mechanisms underlying the development of PAH, mortality rates remain high and available treatments cannot reverse vessels wall injuries. Other treatments targeted on the pathogenesis are being studied. It will be necessary, in the coming years, to develop their clinical utilization in order to improve prognosis of patients with PAH.

Right ventricular dysfunction, mostly due to right ventricular dilation, is the main cause of death. More and more markers of right ventricular function have been studied, but clinical practices disagree concerning parameters that should be used. Currently, hemodynamic values, obtained by cardiac catheterization, remain decisive to establish, not only the diagnosis but also the prognosis, and to follow the evolution of the disease. It is recommended to associate biological, hemodynamic, and imagery parameters. However, it will be necessary to standardize patients’ care and to determine prognostic values of those parameters, in order to optimize follow-up and allow a better monitoring of right heart function.

Finally, nuclear cardiac imaging has been recently used to assess cardiac repercussions of PAH. Number of evidences demonstrated that metabolic abnormalities are present in the right myocardium and that they can be observed by ^18^F-FDG-PET/CT. Their participation in right heart dysfunction has also been highlighted. Even though these first results showed a good sensitivity to assess ventricular metabolism and function, further studies are necessary to confirm its usefulness and its adequacy for clinical patients’ follow-up.

## Author Contributions

This article has been written mainly by CS, under supervision of Prof. LN, JP, and J-DA. Dr. PY and Dr. PM helped with information collection.

## Conflict of Interest Statement

The authors declare that the research was conducted in the absence of any commercial or financial relationships that could be construed as a potential conflict of interest.
